# A Case Series Based on the Mixed Double-Embryo Transfer (MDET) Outcome in Patients With Recurrent Implantation Failure

**DOI:** 10.7759/cureus.53559

**Published:** 2024-02-04

**Authors:** Shilpa Dutta, Akash More, Sanket Mahajan, Neha Nawale, Deepti Shrivastava, Namrata Choudhary

**Affiliations:** 1 Clinical Embryology, Datta Meghe Institute of Higher Education and Research, Wardha, IND; 2 Obstetrics and Gynaecology, Jawaharlal Nehru Medical College, Datta Meghe Institute of Higher Education and Research, Wardha, IND

**Keywords:** female fertility, infertility treatment, assisted reproductive technology (art), endometrial receptivity assay, reproductive health, in vitro fertilization, pregnancy, mdet, infertility, woi

## Abstract

Successful implantation of embryos depends on the synchronous cross-talks between the endometrial wall and the competent blastocyst within the window of implantation (WOI). Hence, the WOI has a major significance in assisted reproductive technology (ART). However, in some cases, women do not have fixed WOI in ART cycles in order to enhance the rate of successful clinical pregnancy. However, there have been stances where women do not have a fixed WOI, and it shifts in subsequent menstrual periods. This contributes to the chances of recurrent implantation failure (RIF). Another factor that contributes to RIF is erratic endometrial receptivity, which hinders the chances of successful implantation of the conceptus in the endometrium. This case series consists of four case studies where the patients were believed to be suffering from RIF due to variable WOI or erratic endometrial receptivity and the routine protocol followed nowadays failed to make them conceive. In order to resolve the condition, we proposed a novel strategy in an attempt to improve pregnancy rates in these cases. An innovative method of embryo transfer known as mixed double-embryo transfer (MDET), which involved the transfer of one day 3 embryo and one day 5 blastocyst on day 6 of progesterone, led to possible pregnancy outcomes. A viable pregnancy was validated based on the human chorionic gonadotropin (β-hCG) test report, and two of the cases delivered healthy babies. Thus, this case series provides a unique approach to addressing the issues of RIF. However, larger studies are required to validate the possible use of this technique.

## Introduction

Infertility has been long defined, according to the guidelines of the World Health Organization (WHO), as the inability to achieve pregnancy despite having regular unprotected sexual intercourse for a period of more than a year [1]. Assisted reproductive technology (ART) has come as a saviour to sort out the problems of infertility. Implantation serves as a key factor phenomenon, which comprises steps in which the embryo gets embedded in a maternal endometrium [2]. A common cause of infertility is implantation failure. It refers to the failure of the embryo to get ensconced into the endometrium due to aberrative causes such as endometrial infections, uterine abnormalities, immunological factors, etc. [2,3]. Implantation depends on the synchronous affinity between the embryo and the endometrium, and there is a specific timeframe during the menstrual cycle when the endometrium is highly receptive for the receival of the embryo. This timeframe is known as the window of implantation (WOI) [4]. It has been found that this period generally occurs between days 20 and 24 in a typical 28-day menstrual cycle [5]. Hence, it is imperative in ART to identify WOI to increase the chances of successful in vitro fertilisation (IVF) pregnancy. Following the transfer of three or more high-quality embryos, the absence of successful progression to clinical pregnancy due to implantation failure is characterized as recurrent implantation failure (RIF) [2]. Many researchers have found that variable WOI is a cause of RIF in at least 25% of the cases [6]. More than 30% of the incidences of RIF have been attributed to erratic endometrial receptivity [6]. This case series comprises four cases that underwent a relatively new procedure known as the mixed double-embryo transfer (MDET) method aimed to tackle the condition of RIF due to variable WOI and erratic endometrial receptivity, resulting in successful clinical pregnancy. To our knowledge, only one case report and one randomised controlled trial on MDET are available in the existing literature. Stamenov et al. were the first pioneer researchers to report a case report on MDET which showed positive outcomes over conventional techniques used [6]. They also conducted a randomised controlled trial including 104 women known to be suffering from RIF, where half of the population underwent MDET over blastocyst double-embryo transfer (BDET). The trial also reported significantly improved outcomes over BDET [7].

## Case presentation

Case 1

**Figure 1:**
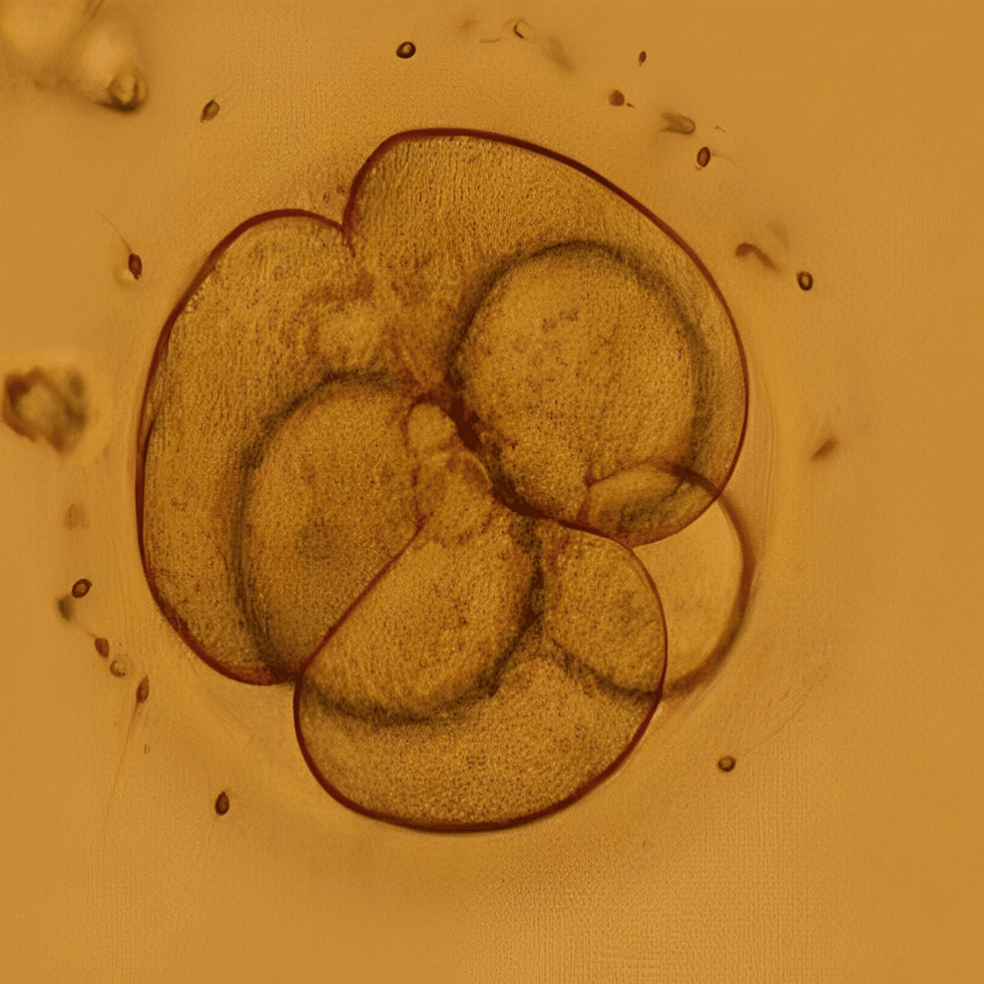
Day 3 embryo Image of the day 3 cleavage stage embryo transferred to the patient in case 1 on day 6 of progesterone

**Figure 2:**
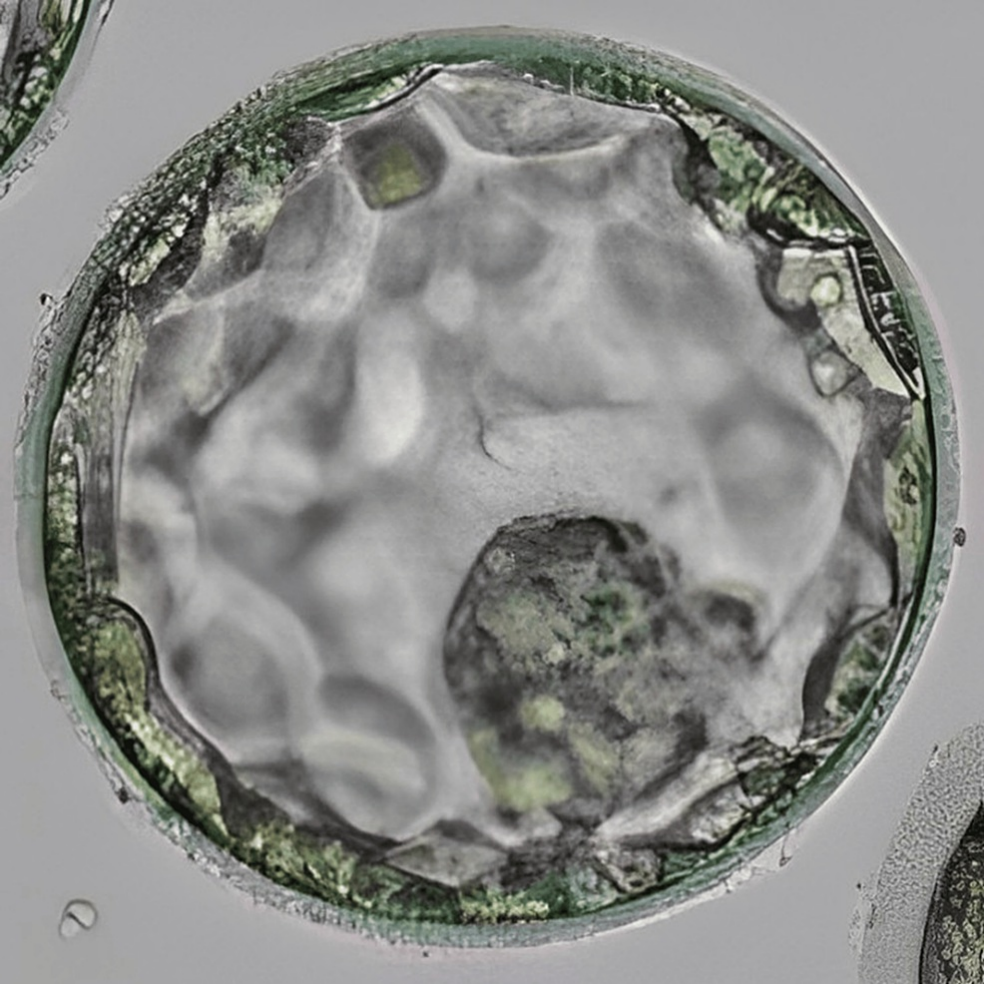
Blastocyst (4AA) Image of the blastocyst (4AA) transferred to the patient in case 1 on day 6 of progesterone

In an intricate case of RIF, a 33-year-old woman and 37-year-old man, married for the last seven years, sought assistance at our infertility clinic after having four IVF failed cycles and six failed embryo transfer cycles without achieving the ultimate goal of conception. Her medical records showed that the woman was nulliparous, but underwent one abortion in her 20s and also had her one oviduct ligated due to ectopic pregnancy caused by the IVF cycle. She also has an average fluctuating menstrual cycle. Her blood report showed a normal range of follicle-stimulating hormone (FSH), luteinizing hormone (LH), and anti-Müllerian hormone (AMH). A good level of AMH indicates good ovarian reserve. The semen analysis report of the male partner indicated him to be normozoospermic. Hysteroscopy was recommended, and the transcript showed all normal parameters of the uterus. This prognosis led to a possible diagnosis of an underlying issue with endometrial receptivity. Conventional IVF was performed for her first cycle at our centre; however, the result was negative. The patient was diagnosed as a case of RIF caused by suspected defective endometrial receptivity. After the counselling of the couple, the treatment plan started with the initiation of antagonist protocol on day 3 of menstrual bleeding. Human menopausal gonadotropin (HMG) at 150 IU daily for seven days was administered, with cycle monitoring every one to two days. Gonadotropin-releasing hormone (GnRH) antagonist cetrorelix was introduced in the presence of follicles with a diameter exceeding 12 mm or LH levels surpassing 10 IU/L. The final maturation of oocytes was induced with 0.2 mg GnRH agonist triptorelin when five dominant follicles reached a diameter greater than 18 mm. Transvaginal ultrasound (TVS)-guided oocyte retrieval was performed 35 hours post oocyte triggering, resulting in the retrieval of seven metaphase II (MII) oocytes. Intracytoplasmic sperm injection (ICSI) was performed, and embryos were frozen on both day 3 (two embryos) and day 5 (five embryos). To further investigate endometrial receptivity, an endometrial receptivity array (ERA) test was conducted on the subsequent menstrual cycle, revealing receptivity on day 19 of the menstrual cycle. Despite sequential embryo transfer (SET) of one cleavage stage embryo and blastocyst each on day 3 and day 6 of progesterone in the subsequent cycle, no pregnancy occurred. In the next cycle, employing the same stimulation protocol, the patient underwent a transfer of two embryos (one on day 3 and one on day 5) on day 6 of progesterone. Figure 1 represents the cleavage stage embryo transferred to the patient, and Figure 2 shows the blastocyst transferred to the patient. This time, the result was positive, validated by a positive human chorionic gonadotropin (β-hCG) test report and observation of the fetal pole via TVS, marking a successful pregnancy.

Case 2

In another intricate scenario, a 36-year-old patient along with her 42-year-old husband who visited our infertility clinic seeking infertility treatment having a medical history of irregular menstrual periods, varying between 28 and 35 days, was identified as a RIF case due to having previous records of four intrauterine insemination (IUI) failed cycles and five ICSI failed cycles. The patient, characterized by a body mass index (BMI) to be 23.5 kg/m² and who reportedly had normal serum FSH levels (FSH: 10 IU/L) on day 2 of the menstrual cycle, underwent controlled ovarian stimulation following a GnRH antagonist protocol. The stimulation involved the administration of follitropin alfa at a dose of 200 IU/day from day 2 of the menstrual cycle. GnRH antagonist (cetrorelix acetate 0.25 mg/day) was administered to the patient when the maturing follicle reached a diameter of 14 mm, continuing until ovulation induction. The ovulation trigger involved the administration of 5000 IU of recombinant hCG when three or more follicles exceeded 17 mm in diameter.

**Figure 3:**
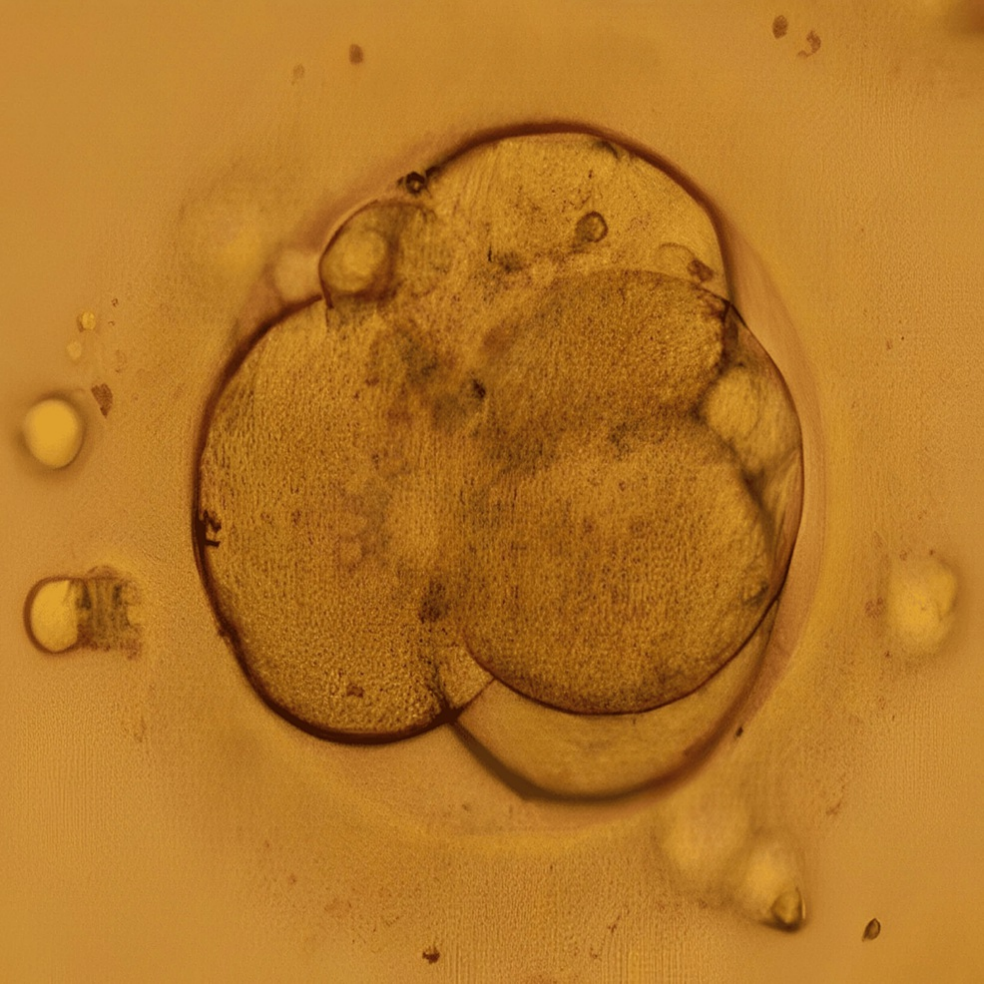
Day 3 embryo Image of the day 3 cleavage stage embryo transferred to the patient in case 2 on day 6 of progesterone

**Figure 4:**
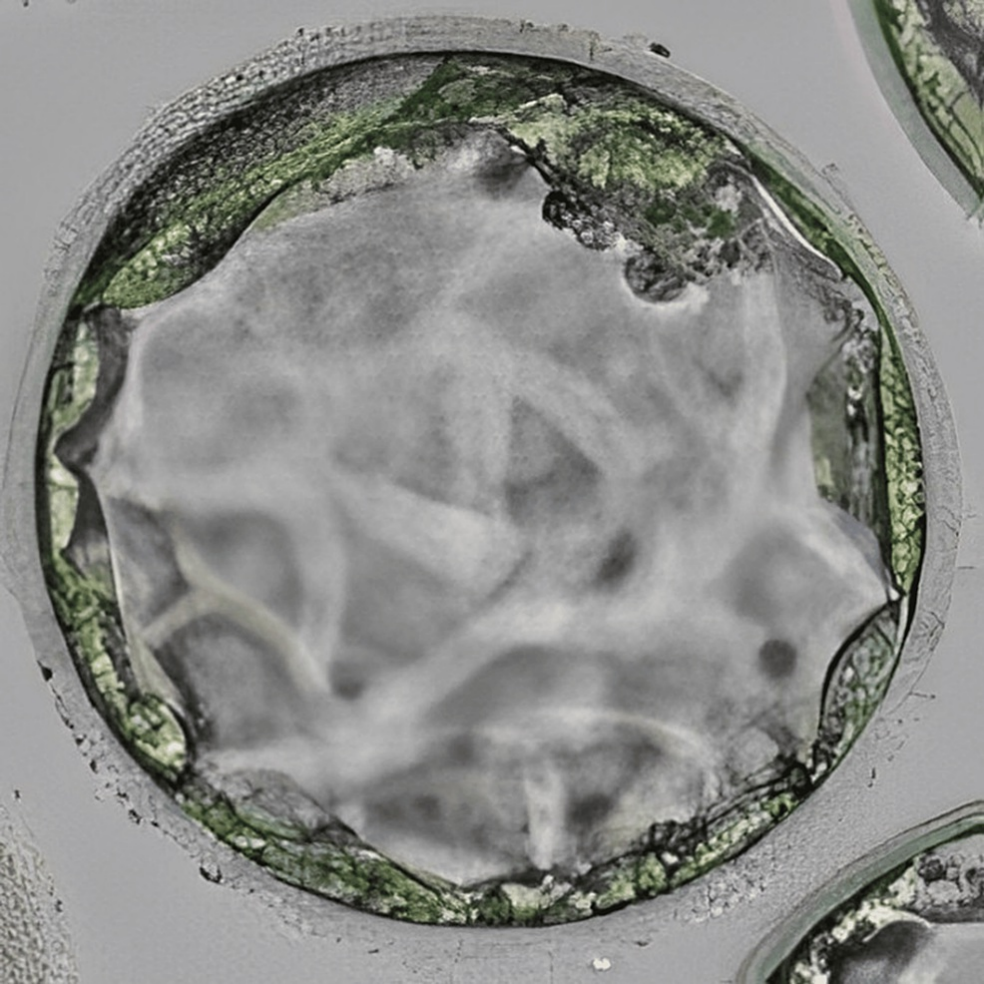
Blastocyst (4BB) Image of the blastocyst (4BB) transferred to the patient in case 2 on day 6 of progesterone

Following the conduction of the first oocyte pick-up (OPU), yielding five oocytes that were subsequently fertilised via ICSI and cryopreserved on day 3 (two embryos) and day 5 (two embryos), one of the embryos underwent arrest in the middle of the embryo culture. ERA test was recommended to the patient and performed for successive three months which revealed fluctuating heightened receptivity between days 23 and 25 of the menstrual cycle. In the following month, a frozen embryo transfer (FET) cycle was undertaken on day 24, involving the preparation of endometrial lining which included oral intake of 6 mg/day estradiol valerate from the third day of the menstrual cycle for 14 days, along with progesterone (400 mg) from five days before embryo transfer. There was a transfer of two blastocysts; however, there were no signs of implantation. Undeterred, the patient underwent another FET in the subsequent cycle on day 23 of the menstrual cycle, incorporating one day 3 embryo and one day 5 blastocyst. Figure 3 represents the cleavage stage embryo transferred to the patient, and Figure 4 shows the blastocyst transferred to the patient. This time, the outcome was a positive pregnancy concluded from the β-hCG value of 1065 mIU/ml. 

Case 3

**Figure 5:**
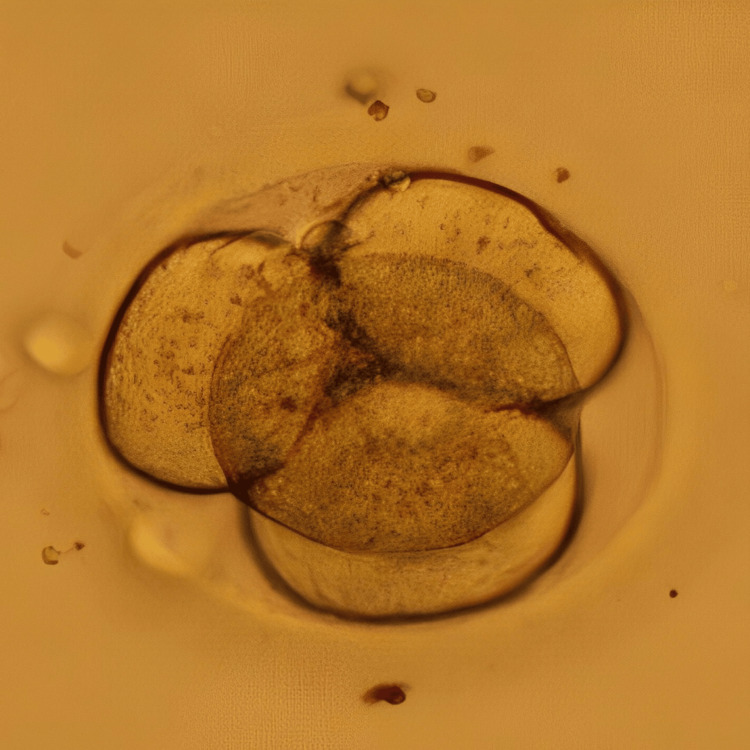
Day 3 embryo Image of the day 3 cleavage stage embryo transferred to the patient in case 3 on day 6 of progesterone

**Figure 6:**
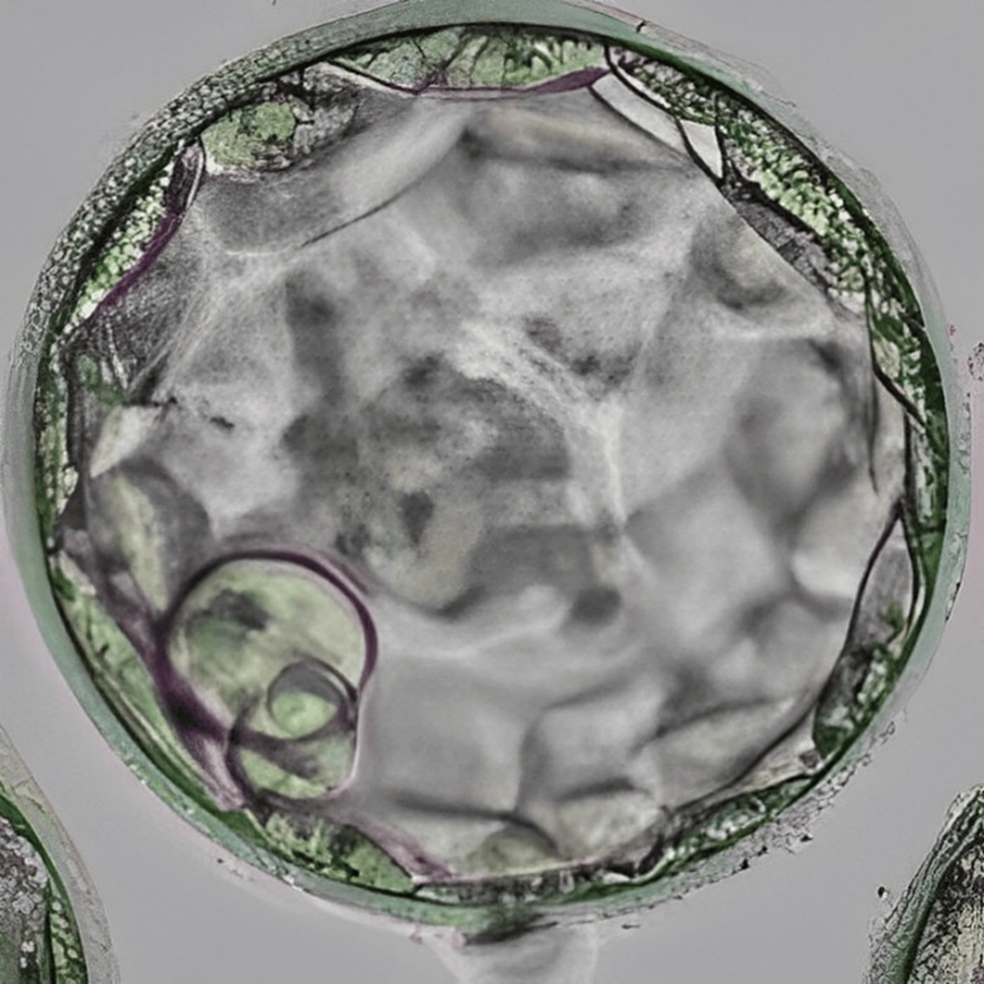
Blastocyst (4AB) Image of the blastocyst (4AB) transferred to the patient in case 3 on day 6 of progesterone

In our reproductive fertility clinic, we encountered a relatively challenging presentation of a 31-year-old woman and 36-year-old man, having a history of two IUI and four ICSI failed cycles and one miscarriage, having secondary infertility, and seeking our consultation. Based on her previous medical records, this case was diagnosed as RIF. The patient had a fluctuating menstrual cycle ranging from 28 days to 34 days in subsequent months. No abnormalities were observed in the findings of hysteroscopic analysis. The semen reports of the partner indicated no abnormalities. Furthermore, both partners underwent chromosomal analysis, revealing normal results. The couple's persistent struggle with infertility prompted a personalised treatment plan. The blood serum analysis shows FSH level to be 7 IU/L, LH to be 4.5 IU/L, and antral follicle count (AFC) to be 12. Semen analysis of the male showed normal parameters. At first, we planned for conventional IVF treatment. GnRH agonist long protocol was followed for the controlled ovarian stimulation. Around 0.5 mg of GnRH was administered for a period of 10-14 days following which the dosage was reduced to 0.2 mg and it was continued till hCG. From day 14, the FSH 200 IU dosage was commenced. Ovarian aspiration was scheduled when the follicles reached >14 mm in diameter. A 5000 IU hCG was administered as a trigger, and oocytes were retrieved 36 hours post trigger. Eight oocytes were retrieved which were fertilised via ICSI. Two embryos were cultured till day 3 and frozen, while on day 6 of progesterone, two blastocysts were transferred to the patient, keeping the rest vitrified. After a fortnight, the pregnancy test resulted in negative. Endometrial scratching was planned as the next mode of treatment for the patient as endometrial scratching has been known to trigger the injured area to repair the site by releasing chemicals which may make the endometrium more receptive towards the conceptus with negligible reports of injections, bleeding, etc. In the non-transfer cycle, the endometrium was scratched on day 1 of hysteroscopy and day 24 of the non-transfer cycle. FET was performed using two blastocysts in the subsequent menstrual cycle, but its result was also negative. In the next cycle, the endometrium was prepared using progesterone five days prior to FET and estradiol 5 mg/day from the second day to the 12th day of the menstrual cycle. One embryo of day 3 and day 5 was thawed and transferred in the uterus transvaginally on day 6 of progesterone. Figure 5 represents the cleavage stage embryo transferred to the patient, and Figure 6 shows the blastocyst transferred to the patient. Fourteen days following the transfer, the blood report of the patient showed positive clinical pregnancy, validated by a β-hCG value of 865 mIU/ml. The patient delivered a healthy boy, weighing 3.2 kg after 38.4 weeks.

Case 4

**Figure 7:**
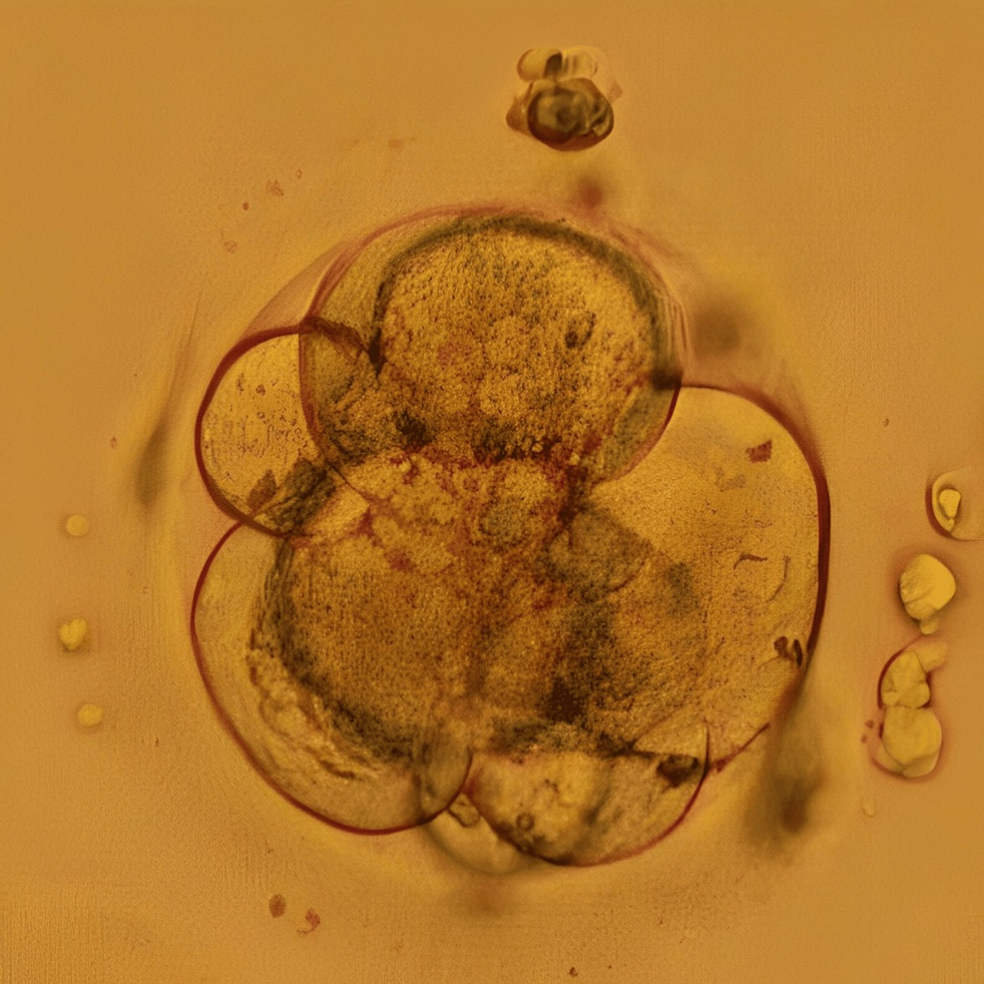
Day 3 embryo Image of the day 3 cleavage stage embryo transferred to the patient in case 4 on day 6 of progesterone

**Figure 8:**
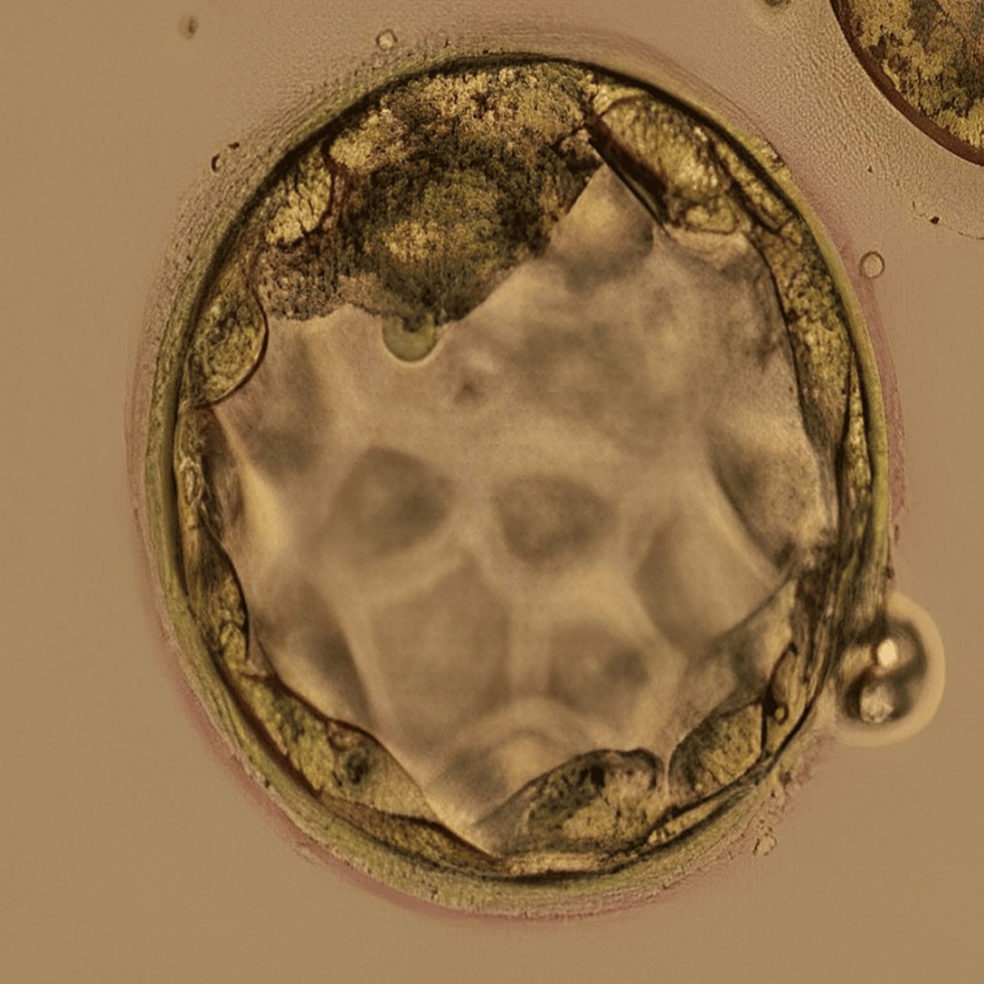
Blastocyst (4AA) Image of the blastocyst (4AA) transferred to the patient in case 4 on day 6 of progesterone

There had been a challenging case presented at our infertility centre, involving a 38-year-old female along with her 41-year-old male partner, having primary infertility in the last 10 years out of their 11-year-old married life. They also had a clinical history of five ICSI failed cycles along with three IUI failed cycles in different centres prior to their visit to our centre. Physical and blood work of both partners showed no signs of abnormalities, with the female having normal blood reports of FSH, LH, and AMH. The male was also having normal semen parameters. A hysteroscopy was also performed on the patient, and it revealed normal physiology of the uterine structure. Hysterosalpingography also revealed normal physiological structures of ovaries and oviducts. However, based on the patient's medical history, she had an average fluctuating range of menstrual cycle ranging from 26 days to 35 days with heavy menstrual bleeding. Also, the patient had a surgical history of removal of uterine fibroid via myomectomy six years prior to seeking infertility treatment. To rule out the cause of endometrial aberration, an endometrial biopsy was performed, and the report pointed out that the patient had erratic endometrial receptivity. According to the guidelines, this case was marked as a RIF case, having the reason for erratic endometrial receptivity. A personalised treatment plan was devised for the patient involving the instillation of hCG in the uterus as hCG is reportedly known to improve endometrial receptivity. Controlled ovarian stimulation was done using GnRH agonist long protocol along with recombinant follicle-stimulating hormone (rFSH). From day 2 of the menstrual cycle, the patient was administered 3.75 mg of GnRH along with 250 mg rFSH till the time there was presentation of three or more maturing follicles measuring above 17 mm. The final trigger was provided using 10,000 IU of subcutaneous hCG injection followed by eight oocyte retrieval post 34.5 hours of the trigger. They were fertilised via ICSI and vitrified on day 3 (two embryos) and day 5 (six embryos). FET cycle was followed in the subsequent month using progesterone in the luteal phase of the menstrual cycle, having instillation of hCG mixed with normal saline intrauterine cavity three days prior to embryo transfer, followed by the transfer of two blastocysts on day 22 of the menstrual cycle. However, the result was negative. The patient was counselled and underwent another round of embryo transfer cycle having transfer of one day 3 embryo and one day 5 embryo on day 6 of progesterone. Figure 7 represents the cleavage stage embryo transferred to the patient, and Figure 8 shows the blastocyst transferred to the patient. A β-hCG test report after a fortnight pointed towards a positive clinical pregnancy. After 38 weeks, the patient delivered a healthy baby girl weighing 2.9 kg.

## Discussion

There is still an ongoing debate as to which stage of the embryo should be transferred in an embryo transfer cycle that may improve the success rate of conception. Nowadays, it has been majorly reported by several prospective trials that day 5 embryo (blastocyst) transfer has a higher conception rate than day 3 embryo transfer [8-10]. Conversely, contemporary conversations highlight potential drawbacks associated with blastocyst transfer. These include a decrease in cumulative live birth rates per couple, an elevated risk of preterm birth, delivering larger-than-average infants for the gestational age, an increased likelihood of monozygotic twins, and a higher incidence of congenital anomalies when compared to embryo transfer during the cleavage stage [6]. Upon reviewing the literature, there has been research based on sequential embryo transfer in which one day 3 embryo is transferred on day 3 and one blastocyst is transferred on day 5/6 of progesterone in a single menstrual cycle [11,12]. Though initial reports suggested improved pregnancy outcomes using sequential embryo transfer, recent research points out that it has a comparable outcome over blastocyst transfer [11-13]. However, it does show superior outcomes over the cleavage embryo transfer procedure [14]. However, there has been only one case report pioneering the idea of MDET using day 3 and day 6 embryos, resulting in addressing the enigma of RIF [6]. Stamenov et al.'s case report was the first pioneer research that has shown positive outcomes using the MDET method over SET [6].

Successful implantation is the culmination of synchronous cross-talks between the embryo and the uterine lining during invagination into the endometrial layer [15]. Hence, it is imperative in ART to identify the correct period of WOI to enhance the chances of a successful pregnancy. It has been majorly noted that in the case of RIF, the primary causative factor behind it has been a variable WOI in consecutive menstrual cycles and erratic endometrial receptivity, which hinders the uterus from receiving the conceptus attachment to it. Different processes are made for the identification of the receptivity of the endometrium, like the ERA test. However, the result isn't guaranteed to be the same in the subsequent cycles, as seen in Stamenov et al.'s case [6]. Although SET seems to provide significant enhancement of pregnancy outcome as compared to blastocyst or cleavage embryo transfer, MDET substantially provides an advantage over SET. It involves only one embryo transfer procedure over two procedures in the case of SET and also addresses problems where the endometrium is pre-receptive on conventional transfer day. It has been majorly found that the reason for implantation failure in RIF has been attributed to the pre-receptivity of the endometrium on conventional transfer day [16].

Limitations

This retrospective investigation encompasses four cases. To substantiate the conceptual framework, imperative next steps should involve the initiation of a randomised clinical trial targeting women experiencing RIF. Participants would be randomly assigned to undergo either the conventional approach (day 5 blastocyst transfer) or the experimental method (combined day 3 and day 5 embryo transfer).

## Conclusions

In a nutshell, this case series seems to be in line with the result of Stamenov et al.'s case report that MDET may provide an alternate significant advantage over conventional protocols that are followed currently in enhancing the rate of pregnancy outcome in patients suffering from RIF due to variable WOI or erratic endometrial receptivity and having good ovarian reserve. However, there is a paucity of data on larger cohorts. Further studies are recommended based on a larger sample targeted population to validate the result of this study.
